# Mr. John Bart Rous

**Published:** 1910-07-23

**Authors:** 


An inquest was held at Westgate last week on Mr. John Bart Rous, who died from morphia poisoning at the age of thirty-one. Mr. Rous, who was on the editorial staff of the *Lancet*, took his diploma in 1903, having studied at St. Mary's Hospital. Here he was afterwards house physician, having graduated M.B., B.S., with honours at London University in 1904. His appointments included that of R.M.O. at Hampstead General Hospital and of house surgeon to the East Sussex Hospital. Then he devoted himself to medical journalism. Recently he had resigned from the R.A.M.C. owing to ill-health. A verdict of "Suicide during temporary insanity" was returned.

